# Doxorubicin-Resistant TNBC Cells Exhibit Rapid Growth with Cancer Stem Cell-like Properties and EMT Phenotype, Which Can Be Transferred to Parental Cells through Autocrine Signaling

**DOI:** 10.3390/ijms222212438

**Published:** 2021-11-18

**Authors:** Anjugam Paramanantham, Eun-Joo Jung, Hye-Jung Kim, Bae-Kwon Jeong, Jin-Myung Jung, Gon-Sup Kim, Hong-Soon Chan, Won-Sup Lee

**Affiliations:** 1 Departments of Internal Medicine, Institute of Health Sciences, Gyeongsang National University Hospital, Gyeongsang National University College of Medicine, 90 Chilam-dong, Jinju 660-702, Korea; anju.udhay@gmail.com (A.P.); eunjoojung@gnu.ac.kr (E.-J.J.); 2 School of Veterinary and Institute of Life Science, Gyeongsang National University, 900 Gajwadong, Jinju 660-701, Korea; 3 Departments of Pharmacology, Institute of Health Sciences, Gyeongsang National University Hospital, Gyeongsang National University School of Medicine, Jinju 660-702, Korea; curlysookim@hanmail.net; 4 Departments of Radiation Oncology, Institute of Health Sciences, Gyeongsang National University Hospital, Gyeongsang National University College of Medicine, 90 Chilam-dong, Jinju 660-702, Korea; blue129j@hanmail.net; 5 Departments of Neurosurgery, Institute of Health Sciences, Gyeongsang National University Hospital, Gyeongsang National University College of Medicine, 90 Chilam-dong, Jinju 660-702, Korea; gnuhjjm@gnu.ac.kr; 6 Department of Surgery, Institute of Health Sciences, Gyeongsang National University Hospital, Gyeongsang National University School of Medicine, Jinju 660-702, Korea; hongsc@gnu.ac.kr

**Keywords:** doxorubicin-resistant, MDA-MB-231, breast cancer, CSCs, EGFR

## Abstract

Emerging evidence suggests that breast cancer stem cells (BCSCs), and epithelial–mesenchymal transition (EMT) may be involved in resistance to doxorubicin. However, it is unlear whether the doxorubicin-induced EMT and expansion of BCSCs is related to cancer dormancy, or outgrowing cancer cells with maintaining resistance to doxorubicin, or whether the phenotypes can be transferred to other doxorubicin-sensitive cells. Here, we characterized the phenotype of doxorubicin-resistant TNBC cells while monitoring the EMT process and expansion of CSCs during the establishment of doxorubicin-resistant MDA-MB-231 human breast cancer cells (DRM cells). In addition, we assessed the potential signaling associated with the EMT process and expansion of CSCs in doxorubicin-resistance of DRM cells. DRM cells exhibited morphological changes from spindle-shaped MDA-MB-231 cells into round-shaped giant cells. They exhibited highly proliferative, EMT, adhesive, and invasive phenotypes. Molecularly, they showed up-regulation of Cyclin D1, mesenchymal markers (β-catenin, and N-cadherin), MMP-2, MMP-9, ICAM-1 and down-regulation of E-cadherin. As the molecular mechanisms responsible for the resistance to doxorubicin, up-regulation of EGFR and its downstream signaling, were suggested. AKT and ERK1/2 expression were also increased in DRM cells with the advancement of resistance to doxorubicin. Furthermore, doxorubicin resistance of DRM cells can be transferred by autocrine signaling. In conclusion, DRM cells harbored EMT features with CSC properties possessing increased proliferation, invasion, migration, and adhesion ability. The doxorubicin resistance, and doxorubicin-induced EMT and CSC properties of DRM cells, can be transferred to parental cells through autocrine signaling. Lastly, this feature of DRM cells might be associated with the up-regulation of EGFR.

## 1. Introduction

Cancer is one of the most fatal diseases in the world, and breast cancer is one of the most frequent cancer types of cancer in women in terms of incidence and mortality [1]. Breast cancer is a highly heterogeneous disease that displays diverse morphological features and variable clinical outcomes [2]. The responsiveness to treatment of breast cancer is dependent on their specific biological characteristics. Therefore, some breast cancer classifications have been developed [3]. A breast cancer classification includes basal-like, luminal A or B, luminal ER−/AR+, and ERBB2/HER2-amplified. Among them, basal-like, also called triple-negative, breast cancer (TNBC) shows more aggressive behaviors (higher grade, proliferation, and recurrence rate) than other types of breast cancer [4]. The reason why it is called TNBC is that it tests negative for estrogen receptors, progesterone receptors, and excess ERBB2/HER2 protein, which means that it does not respond to hormonal or HER2-targeted therapy [5]. For TNBC, the main treatment contains conventional cytotoxic systemic chemotherapy. Initially, TNBC is susceptible to conventional chemotherapy, but the initial susceptibility to treatment does not correlate with overall survival even in patients with TNBC who obtained complete remission [6,7,8,9].

Among systemic chemotherapeutic agents, doxorubicin is one of the most frequently used drugs [10]. It induces DNA intercalation, topoisomerase II inhibition and free radical formation [11]. It is well recognized that longer exposure to chemotherapeutic agents may generate an adaptive cellular response that results in the induction of acquired drug resistance [12]. TNBC usually acquires resistance to doxorubicin; after acquiring resistance to doxorubicin, the cancer cells develop multi-drug resistant phenotypes [13,14].

Recently, emerging evidence suggests that breast cancer stem cells (BCSCs), and epithelial-mesenchymal transition (EMT) may be involved in doxorubicin resistance [15].

Cancer stem cells (CSCs) can extensively proliferate, self-renew, differentiate to multiple lineages and generate a tumor mass [16]. TNBC is enriched in cancer stem cell populations, and CSCs are distinguished from other cancer cells by the expression of cell surface markers CD44^+^/CD24^−^ [4,17] and overexpression of octamer-binding transcription factor 3/4 (OCT-3/4) [18]. Recent studies provide clear evidence that breast cancer stem cells have a highlighted role in recurrence and distant metastasis as well as drug resistance [19,20,21].

EMT is the process that involves a loss of epithelial features with acquiring mesenchymal features, and acquisition of enhanced invasive and metastatic behaviors. It is also involved in enhanced cancer cell survival, and immune tolerance. In addition, the activation of EMT programs in cancer cells expands their generation of chemo-resistant breast cancer stem cells (BCSCs). Many signaling pathways such as TGF-β, Wnt, Notch, TNF-a, NF-кB, RTK, MAPK/ERK, and PI3K/Akt are involved in CSCs maintenance [22,23,24]. Several lines of evidence suggest that CSCs generated through EMT exhibit resistance to conventional chemotherapies [22]. EMT and CSCs are also deeply involved in doxorubicin resistance through dormancy [15,25]. Therefore, it is believed that activation of EMT programs is tightly linked with the expansion of cancer stem cells [10,26,27] and that CSCs exhibit doxorubicin resistance through dormancy [28].

However, it is uncertain whether doxorubicin-induced EMT and acquisition of CSC properties are related to cancer dormancy or outgrowth of cancer cells with the maintenance of doxorubicin resistance because the phenotype of radio-resistant breast cancer cells showed high proliferative phenotype while harboring the phenotype of EMT and CSCs [29]. In addition, the mechanisms accounting for the maintenance and/or induction of EMT and CSCs still remain largely obscure [10], especially in relation to the transfer of these phenotypes to other doxorubicin-sensitive cells [26].

In this study, we characterized the phenotype of doxorubicin-resistant TNBC cells while monitoring the EMT process and expansion of CSCs during the establishment of mechanisms associated with the EMT process doxorubicin-resistant TNBC cells. In addition, we assessed the potential signaling associated EMT process and expansion of CSCs in doxorubicin-resistant TNBC cells. Targeting the signaling pathways involved in doxorubicin-resistant TNBC cells may improve the effectiveness of therapeutic modalities for TNBC.

## 2. Results

### 2.1. Doxorubicin-Resistant MDA-MB-231 (DRM) Cells Were Established by Continuous Treatment with Increasing Concentrations of Doxorubicin

**Figure 1:**
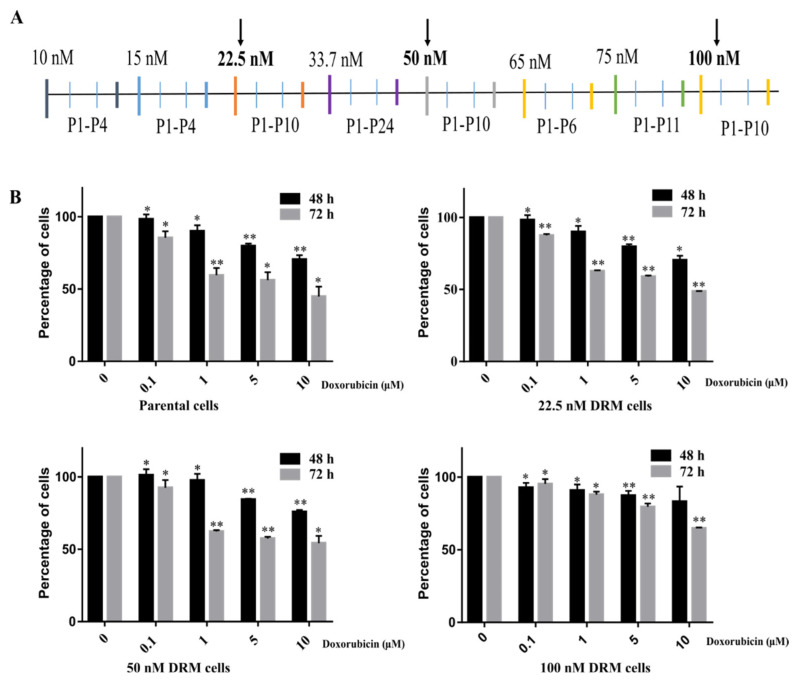
The schedule for the establishment of DRM cells and cell viability of DRM cells with (**A**) MDA-MB-231 human breast cancer cells were treated with doxorubicin with the increasing concentration ranges from 10 nM to 100 nM. Based on the human plasma concentrations of doxorubicin, the final concentration (100 nM) was designed. The cells were treated and maintained with doxorubicin, and the number of passages is represented as P1, P2… Cells were maintained in doxorubicin-containing media for 38 weeks. (**B**) The cell viability assay for the three clones (22.5, 50 and, 100 nM DRM cells) that showed morphological changes while establishing doxorubicin-resistant cells. The cells showed resistance to doxorubicin as compared to parental cells. The values expressed as mean ± standard deviation (SD) (*n* = 5) (* *p* < 0.05 vs. control; ** *p* < 0.001 vs. control). DRM: Doxorubicin-resistant MDA-MB-231.

To establish doxorubicin-resistant cells, we treated them with continuously increasing concentrations of doxorubicin up to 100 nM as the final concentration. The experimental design for the induction of doxorubicin resistance is depicted in Figure 1A. The cells which showed resistance at 22.5, 50 and 100 nM were kept in the liquid nitrogen tank (−190 °C) for further studies. Cells that showed viability with the treatment of 10 μM doxorubicin were considered resistant because the plasma concentration of doxorubicin is ~100 nM [10,30]. The parental MDA-MB-231 cells (p-MDA-MB-231 cells) showed 38% cell viability at 72 h, while 22.5, 50 and, 100 nM DRM cells showed 49%, 54% and, 64% of cell viability at 72 h at 10 μM of doxorubicin. The IC50 value at 72 h of p-MDA-MB-231 cells were 6.5 μM whereas the IC50 values of 22.5, 50 and, 100 nM DRM cells were 8.9 μM, 10.9 μM, and 14.3 μM, respectively. We performed further experiments with 22.5, 50 and, 100 nM DRM cells to characterize the phenotype of doxorubicin-resistant TNBC cells.

### 2.2. DRM Cells Showed Morphological Changes and Increased Proliferative Capacity while Acquiring Resistance to Doxorubicin

**Figure 2:**
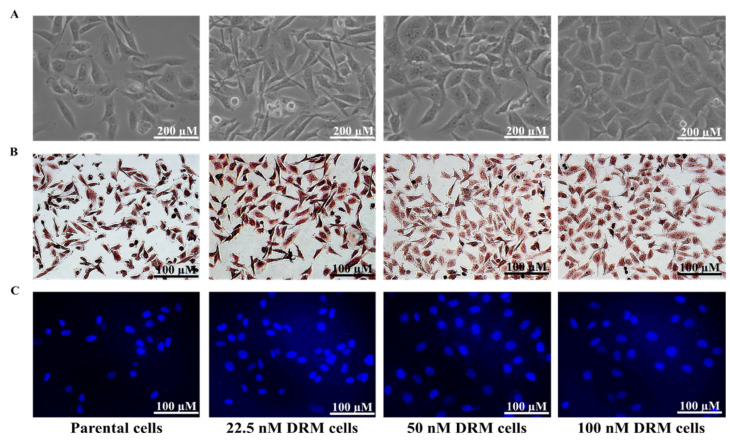
Morphological analysis of DRM cells (**A**) Morphological analysis performed under a light microscope 48 h after cells were seeded. DRM cells showed an increase in cell size and cell-to-cell communication. The cells changed from spindle-like structures to cobblestone-like giant cells as acquiring resistance to doxorubicin (Magnification, ×200; scale bar, 200 µm) (**B**) Whole-cell morphology analyzed with Mayer’s staining. DRM cells showed morphological changes with acquiring resistance to higher concentrations of doxorubicin. (Magnification, ×100; scale bar, 100 µm) (**C**) DAPI staining showing the nuclear morphology of DRM cells. DRM cells showed an increase in nuclear size with advancement in doxorubicin resistance. Results were confirmed by three independent experiments. (Magnification, ×100; scale bar, 100 µm). DRM: Doxorubicin-resistant MDA-MB-231.

The morphological changes in the DRM cells were photographed under a light microscope. The parental cells changed from spindle-like structures to cobblestone-like giant cells as acquiring resistance to doxorubicin (Figure 2A). The proliferative activity was also highly increased in the DRM cells as acquiring resistance to higher concentrations of doxorubicin (Figure 2A). To further characterize the morphological difference between the parental and the DRM cells, we performed Mayer and DAPI staining (Figure 2A,B). DRM cells showed an increase in the number and size of cells while acquiring resistance to doxorubicin. In addition, the Mayer stain also unravels the morphological changes of DRM cells as an advancement of resistance to doxorubicin (Figure 2B). DAPI staining revealed that DRM cells have a larger nuclear size than the parental cells while acquiring resistance to higher concentrations of doxorubicin (Figure 2C). These findings suggest that DRM cells have morphological changes and increased proliferative activity while acquiring resistance to higher concentrations of doxorubicin.

### 2.3. DRM Cells Showed an Increase in Proliferation, Invasion, Migration, and Adhesion Characteristics

**Figure 3:**
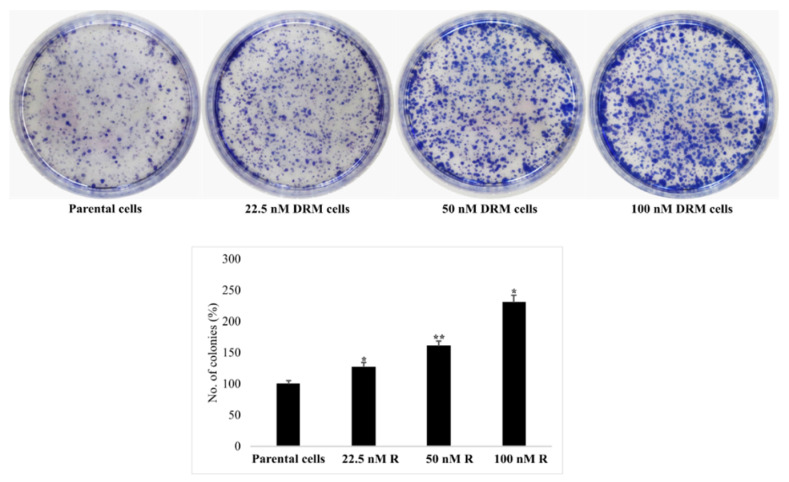
Cell proliferation ability. DRM cells showed high proliferation rates compared to parental cells. Parental MDA-MB-231 and DRM cells were seeded, and media was changed every 3 days. After 10 days, cells were stained with Giemsa stain and photographed. Results were confirmed by three independent experiments. DRM: Doxorubicin-resistant MDA-MB-231. (* *p* < 0.05 vs. control; ** *p* < 0.001 vs. control). DRM: Doxorubicin-resistant MDA-MB-231.

Unexpectedly, DRM cells showed high proliferative activity. Here, we confirmed this finding with a colony-forming assay, which revealed that DRM cells acquired high proliferative ability; the number of colonies increased as an advancement of resistance to higher concentrations of doxorubicin (Figure 3).

**Figure 4:**
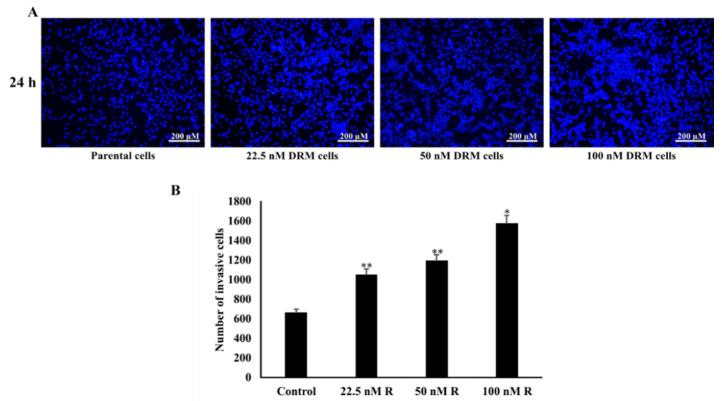
Invasion effect of DRM cells. The transwell invasion assay showed a significant increase in the invasion of DRM cells with the advancement of resistance to higher concentrations of doxorubicin. The cells were seeded with the seeding density of 5 × 10^4^ cells/well to the upper chamber of the Boyden chamber which is pre-coated with Matrigel and the cells invaded through the Matrigel were stained with DAPI. (**A**) The stained cells were photographed and (**B**) counted through ImageJ software and a number of invasive cells were graphed. The values expressed as mean ± standard deviation (SD) (*n* = 5) (* *p* < 0.05 vs. control; ** *p* < 0.001 vs. control). DRM: Doxorubicin-resistant MDA-MB-231.

**Figure 5:**
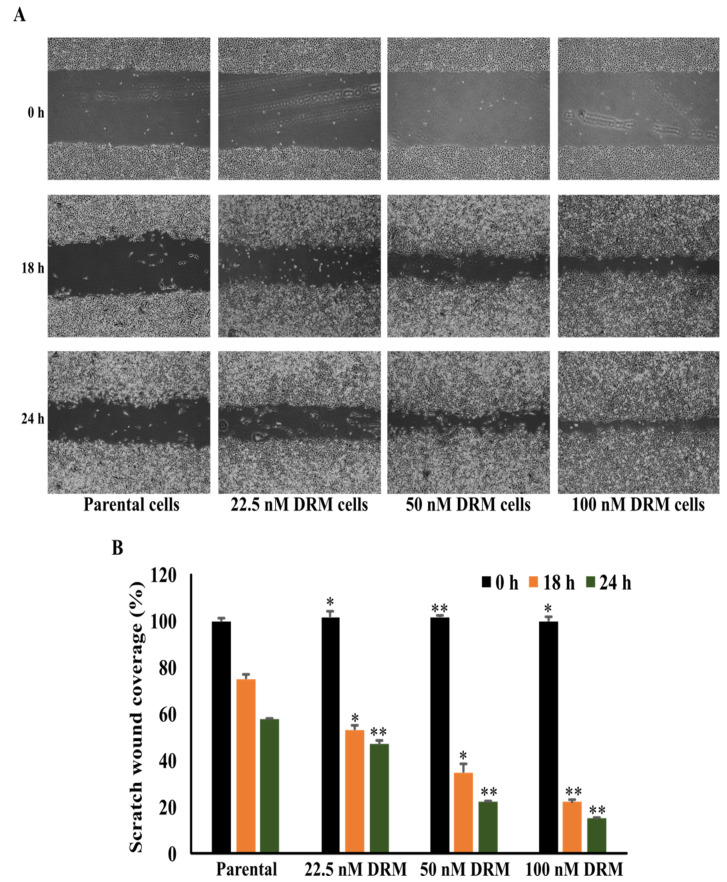
DRM cells have an increased migration potential. The wound healing assay indicated the area of wound closure in 22.5 nM, 50 nM, 100 nM DRM cells, and the parental cells which were 47%, 22%, 15%, and 58%, respectively. The cells were grown with 100% confluence and a wound was created with the use of a 1 mL tip. (**A**) The migratory effect of the cells was analyzed with the photograph of the etched area with the mentioned durations. (**B**) The area of the cell migration was measured with the use of ImageJ software and graphed. The values expressed as mean ± standard deviation (SD) (*n* = 5) ((* *p* < 0.05 vs. prental cells;, ** *p* < 0.001 vs. parental cells). DRM: Doxorubicin-resistant MDA-MB-231.

**Figure 6:**
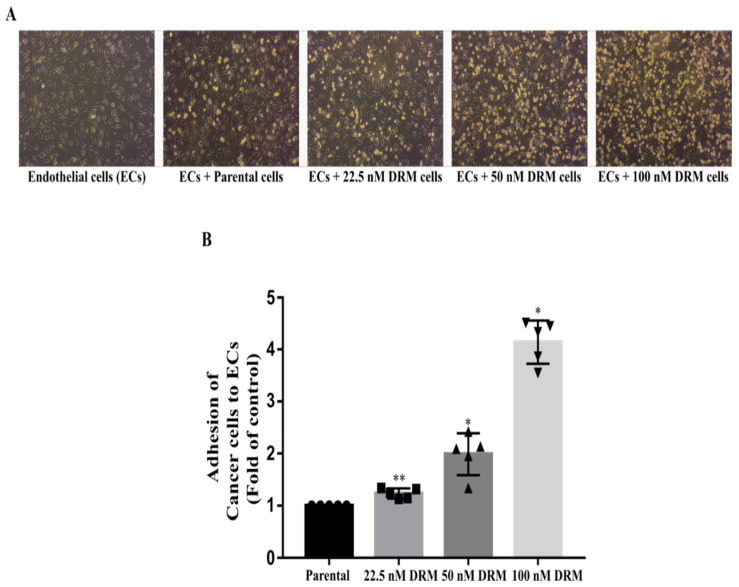
Doxorubicin-resistant cells have a high adhesion effect to umbilical vein endothelial cells (ECs). The adhesion of DRM cells to Endothelial cells (ECs) was significantly increased with the advancement of resistance to higher concentrations of doxorubicin. The cells were washed, and the resistant cells adhered to ECs were counted. (**A**) The attached cells were photographed using a light microscope (**B**) Graphical representation of a number of cells attached. The values expressed as mean ± standard deviation (SD) (*n* = 5) (* *p* < 0.05, **p < 0.001 vs. control). DRM: Doxorubicin-resistant MDA-MB-231.

Resistance to chemotherapy as a result of continuous exposure to the chemotherapeutic drug is usually accompanied by the enhanced migration and metastasis of tumor cells [28]. Therefore, we checked whether DRM cells increased the ability of invasion, migration, and adhesion. The transwell invasion assay showed a significant increase in an invasion of DRM cells with the advancement of resistance to higher concentrations of doxorubicin. The relative invasion ability of the 22.5 nM, 50 nM, 100 nM DRM cells, and the parental cells as control were 151%, 181%, 238%, and 100% at 24 h, respectively (Figure 4A). With regard to the cell migration, the wound healing assay indicated the area of wound closure in 22.5 nM, 50 nM, 100 nM DRM cells, and the parental cells which were 47%, 22%, 15%, and 58%, respectively (Figure 5). The adhesion of DRM cells to endothelial cells (ECs) was significantly increased with the advancement of resistance to higher concentrations of doxorubicin (Figure 6A). These results suggest that DRM cells acquired an increased proliferation, invasion, migration, and adhesion ability, with the advancement of resistance to higher concentrations of doxorubicin.

### 2.4. DRM Cells Expanded the Population of CSCs as Acquiring Resistance to Doxorubicin

**Figure 7:**
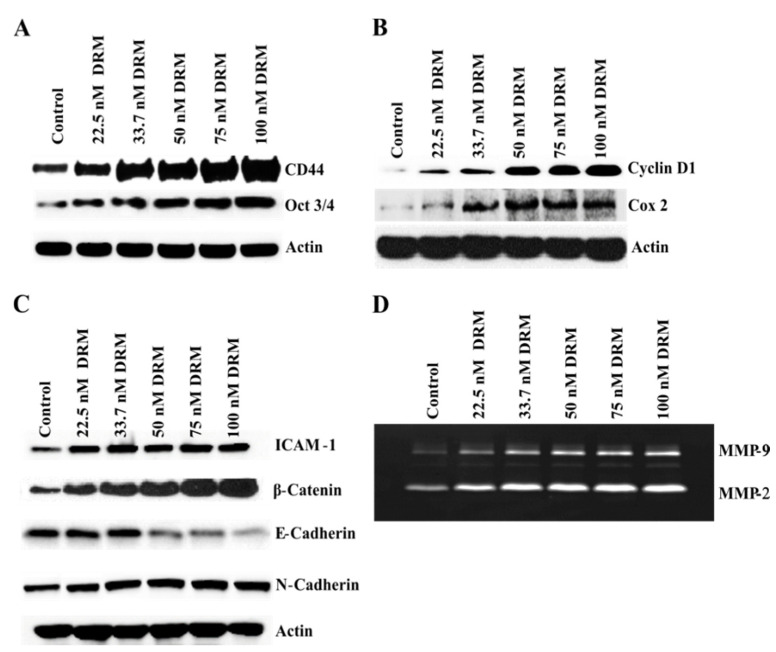
DRM cells showed high cancer stem cells, high proliferation, and high EMT phenotype. Western blot analysis of markers for (**A**) cancer stem cells, (**B**) proliferation (**C**) EMT phenotype related proteins, and (**D**) Gelatin zymography of MMP proteins. Cells were seeded with a seeding density of 5 × 10^4^ cells and grown for 72 h. The control cells were left untreated. The whole-cell protein lysate was prepared, and 30 µg of proteins were resolved in SDS-polyacrylamide gels. Western blots were confirmed by three independent experiments. DRM: Doxorubicin-resistant MDA-MB-231.

Cancer stem cells (CSCs) are highly associated with the development of drug-resistant cancer cells [25]. To test whether CSCs expanded while acquiring doxorubicin resistance, we investigated the expression of CD44, a representative CSC marker [10,30,31,32]. Western blot analysis revealed that DRM cells increased the expression of CD44 by acquiring doxorubicin resistance (Figure 7A). Another CSC marker, OCT 3/4 is important in maintaining the pluripotent cells [33]. The expression was also increased with the advancement of doxorubicin resistance of DRM cells (Figure 7A). These findings suggest that the population of CSC of DRM cells expanded with the advancement of resistance to higher concentrations of doxorubicin.

### 2.5. DRM Cells Showed Highly Proliferative, EMT, Adhesive, and Invasive Phenotypes Molecularly

Next, we tried to molecularly confirm that DRM cells showed an increase in proliferation, invasion, migration, and adhesion characteristics. First, we assessed the expression of Cyclin D1 because it is a representative biomarker for cell proliferation. Western blot analysis revealed that cyclin D1 was up-regulated in DRM cells’ advancement of resistance to higher concentrations of doxorubicin (Figure 7B). Next, we investigated the EMT phenotype because EMT plays a significant role in tumor progression, metastasis, and chemo-resistance [10,34]. Western blot analysis revealed that DRM cells expressed up-regulation of mesenchymal markers like β-catenin, N-cadherin, and down-regulation of epithelial markers like E-cadherin with the advancement of resistance to higher concentrations of doxorubicin (Figure 7C). These findings were consistent with EMT. As an adhesion molecule, we chose the adhesion molecule ICAM-1. It also showed a significant increase in DRM cells as acquiring resistance to higher concentrations of doxorubicin (Figure 7C). MMP-2 (gelatinase-A), and MMP-9 (gelatinase-B) are involved in proteolytic digestion of the extracellular matrix (ECM) for cancer invasion and metastasis [35]. Thus, we investigated the expression of MMP-2 and MMP-9 with gelatin zymography, which showed an increase in MMP-2 and -9 expressions with the advancement of resistance to higher concentrations of doxorubicin (Figure 7D). The molecular expressions of DRM cells suggest that DRM cells acquired proliferation, EMT, adhesion, invasion, and metastasis phenotype.

### 2.6. Epidermal Growth Factor Receptor (EGFR) Upregulation Was Associated with Doxorubicin Resistance of DRM Cells

**Figure 8:**
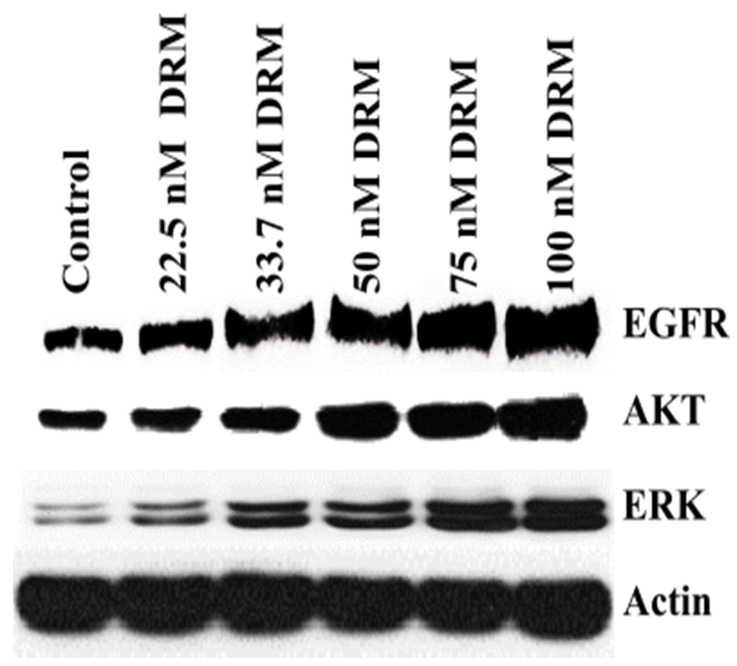
DRM cells showed high EGFR activity. Western blot analysis of EGFR and the downstream signal, Akt and ERK. Cells were seeded with a seeding density of 5 × 10^4^ cells and grown for 72 h. The control cells were left untreated. The whole-cell protein lysate was prepared, and 30 µg of proteins were resolved in SDS-polyacrylamide gels. Western blots were confirmed by three independent experiments. DRM: Doxorubicin-resistant MDA-MB-231.

Up-regulation of the epidermal growth factor receptor (EGFR) is associated with high proliferation and drug resistance [36]. Here, we investigated the expression of EGFR in DRM cells. Western blot analysis revealed that EGFR expression was increased with the advancement of resistance to higher concentrations of doxorubicin (Figure 8). The two representative downstream signals of EGFR, AKT, and ERK 1/2 were also increased in DRM cells with the advancement of resistance to higher concentrations of doxorubicin (Figure 8). These results suggest that the doxorubicin resistance of DRM cells was at least in part involved in the upregulation of EGFR and its activation of downstream signaling.

### 2.7. Doxorubicin Resistance of DRM Cells Can Be Transferred to p-MDA-MB 231 Cells by Autocrine Signaling

**Figure 9:**
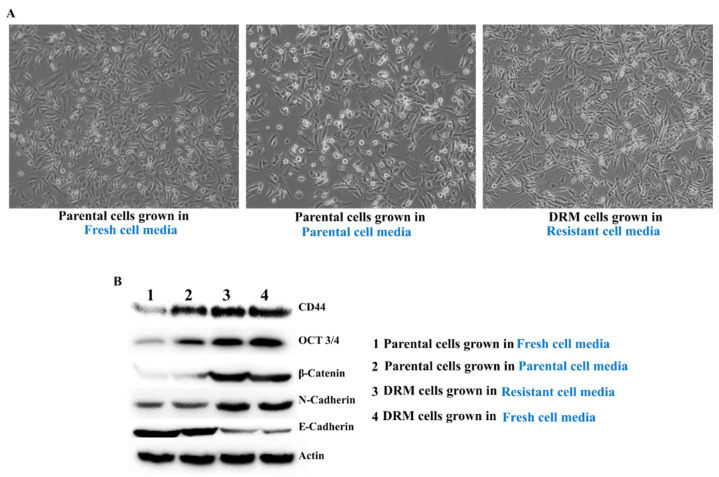
Autocrine signaling in maintaining DRM cells. The parental cells were grown in resistant cell media showed an almost similar pattern of DRM cells in terms of the expression of CSC and EMT phenotype. Parental- MDA-MB-231 cells were grown in Doxorubicin-resistant cells grown media. (**A**) Morphological analysis of Parental- MDA-MB-231 cells, Parental- MDA-MB-231 cells grown in Doxorubicin resistant cells grown media. (**B**) Western blot analysis of autocrine signaling by analyzing the cancer stem cell markers and EMT phenotype. Results were confirmed by three independent experiments. DRM: Doxorubicin-resistant MDA-MB-231.

Cell to cell communication networks have been one of the many driving forces behind the development of drug resistance and CSC [28,37]. Thus, to determine whether autocrine mechanisms are implicated in the tumor microenvironment, we grew the parental cells in the resistant cell-grown media and compared them against cells grown in fresh cell media. The parental cells grown in resistant cell media showed an almost similar pattern of DRM cells in terms of the expression of CSC and EMT phenotype (Figure 9). These results show that the autocrine factors might be important in acquiring doxorubicin resistance of DRM cells and the expansion of the CSC population in DRM cells.

## 3. Discussion

This study was designed to determine the characteristics of DRM cells morphologically and molecularly, and to answer whether DRM cells showed doxorubicin resistance with dormancy and whether the phenotypes can be transferred to other doxorubicin-sensitive cells. This study clearly demonstrated that DRM cells were outgrowing cancer cells with maintaining resistance to doxorubicin, and the EMT features with CSC properties can be transferred to other doxorubicin-sensitive cells through autocrine signaling. In addition, we demonstrated DRM cells changed from spindle-like structures to cobblestone-like giant cells with a larger nucleus and highly proliferative activity. In addition, these cells also acquired highly invasive, migratory, and adhesive abilities. Molecularly, DRM cells exhibited an enrichment of the EMT features with CSC properties. Up-regulation of EGFR might be associated with the establishment of DRM cells.

The morphological changes from spindle-like structures to cobblestone-like giant cells indicated that the DRM cells were undergoing EMT [38]. This process was facilitated by reducing apical-basal polarity and epithelial adhesion proteins [39]. However, DRM cells showed high adhesive ability (Figure 6). In addition, mesenchymal-like cancer cells that have undergone EMT may remain in a dormant state after attaching the metastatic sites [10,26], because of the recurrence, decades after primary tumor resection and adjuvant therapy [40]. However, DRM cells did show rapid growth instead of dormancy. We found that DRM cells have unique features including highly proliferative and adhesive properties with EMT features. This can be explained by other studies that showed hybrid EMT/MET CTCs [10,26]. This model is to explain the features of metastatic cancer cells that do not match the EMT/MET cancer metastasis hypothesis. Some investigators explained this phenomenon with a partial EMT model [41]. EMT is not a dichotomous switch between epithelial or mesenchymal status, but intermediate states; this helps to explain the notion that cancer cells utilize the EMT program for metastasis with dormancy, whereas MET helps establish the metastatic outgrowth [27]. There is another model explaining this finding; the cancer cells acquiring EMT with CSCs have high proliferative activity [26]. This finding is more appealing to us and is consistent with our own findings. In addition, we previously demonstrated that radiation-resistant MDA-MB 231 cells were also highly proliferative while they exhibited an enrichment of the EMT features with CSC properties [42]. All this supports our findings.

Regarding the high adhesive activity firstly, we thought that the downregulation of E-cadherin may contribute to a decrease in adhesive activity, but DRM cells showed high adhesive activity compared to parental cells (Figure 6). Hence, we test the expression of I-CAM that is a highly expressed adhesion protein in highly metastatic cancer cells [43]. DRM cells also showed high expression of I-CAM. This finding also suggests that DRM cells are more similar to highly metastatic cancer cells rather than cancer cells in dormancy. Next, we searched the signaling involved in drug resistance with upregulation of cyclin D1 and I-CAM. We found that up-regulation of EGFR and downstream signaling activation was increased with the advancement of doxorubicin resistance (Figure 8). The up-regulation of EGFR and the downstream signals, Akt and ERK activation induces upregulation of cyclin D1 and I-CAM [36]. In addition, inhibition of EGFR inhibitor, gefitinib can inhibit doxorubicin resistance [44], and increased expression of EGFR may be associated with conferring resistance of doxorubicin [45]. These findings support our findings that DRM cells look like highly metastatic cells with rapid growth.

Lastly, regarding the transmission of doxorubicin resistance of DRM cells to parental cells, it was reported that autocrine signaling is important in inducing and maintaining mesenchymal and CSCs in the breast cancer [46,47]. That is why we tested whether the doxorubicin resistance of DRM cells can be transferred to a parental cell. As shown in this study, we clearly demonstrated that the doxorubicin resistance of DRM cells can be transferred to parental cells through autocrine signaling (Figure 9).

The limitation of this study is that we only performed an experiment with only one cell line. It is still questioned whether this finding can be applied to all doxorubicin-resistant breast cancer cell lines or can be generalized only to triple-negative breast cancer cells. Further research is warranted to answer this question.

In summary, DRM cells were outgrowing parental cells while maintaining resistance to doxorubicin, and the EMT features with CSC properties can be transferred to other doxorubicin-sensitive cells through autocrine signaling. DRM cells changed from spindle-like structures to cobblestone-like giant cells with a larger nucleus, exhibiting increased, invasion, migration, and adhesion ability. They were highly metastatic cancer cells with rapid growth, but they harbored EMT features with CSC properties. Lastly, the feature of DRM cells might be associated with the up-regulation of EGFR.

## 4. Materials and Methods

### 4.1. Cell Culture and Chemicals

The triple-negative human breast cancer cell line MDA-MB-231 was obtained from Korea cell line bank and was sub-cultured with Roswell Park Memorial Institute Medium (RPMI) 1640 media (Hyclone, Marlborough, MA, USA) containing 10% of heat-inactivated (*v*/*v*) FBS (fetal bovine serum) (GIBCO BRL, Grand Island, NY, USA), 1 mM l-glutamine, 100 U/mL penicillin, and 100 μg/mL streptomycin at 37 °C in a humidified atmosphere of 95% air and 5% CO_2_. Antibodies against OCT-3/4, AKT, β-Catenin, ERK ½, ICAM-1 were purchased from Santa Cruz Biotechnology (Santa Cruz, CA, USA). Antibodies against CD44, E-cadherin, N-cadherin, were purchased from Abcam. Antibody against β-actin was purchased from Sigma (Beverly, MA, USA). Peroxidase-labeled donkey anti-rabbit and sheep anti-mouse immunoglobulin, and an enhanced chemiluminescence (ECL) kit were purchased from Amersham (Arlington Heights, IL, USA). All other chemicals not specifically cited here were purchased from Sigma Chemical Co. (St. Louis, MO, USA).

### 4.2. Preparation of Doxorubicin Resistant MDA-MB-231 Cells

The DRM phenotype was established by exposing the cells to doxorubicin in an increasing concentration (10 nM–100 nM). The final concentration of doxorubicin is determined as 100 nM according to the plasma concentration of doxorubicin. The experimental design depicting the treatment of doxorubicin is shown in Figure 1A. The cells were continuously exposed to different concentrations of doxorubicin. The number of passages the cells were maintained in each concentration is depicted in Figure 1A. Considering each passage is for 3 days in total, induction of resistance took about 38 weeks. The cells were considered resistant when there were no dead cells seen. The cells showed resistance at different passages in different concentrations of the drug. Throughout the induction period, the cells at the initial passage and final passage are collected. The same method was also followed for the untreated group to identify the passage-related alterations in the cells. The cells were considered chemo-resistant with the ability to grow in 10 μM of doxorubicin.

### 4.3. Cell Viability Assay

Cells were seeded in 24 well plates with a seeding density of 5 × 10^4^ cells/well. Cells were treated with and without doxorubicin as indicated (0–10 μM). After 48 h and 72 h incubation at 37 °C in CO_2_ incubator the cells were treated with 50 μL of 3-(4,5-dimethylthiazol-2-yl)-2,5-diphenyltetrazolium bromide (MTT) solution (5 mg/mL in 1× PBS) and kept for 3 h at 37 °C in a CO_2_ incubator. After incubation, the supernatant was removed, and the formazan crystals were dissolved with 200 μL of Dimethyl Sulfoxide (DMSO). The absorbance was read at 540 nm on a microplate reader (Bio-Rad, Hercules, CA, USA).

### 4.4. Invasion Assay

To identify the invasiveness of Doxorubicin-resistant cells, we performed a transwell assay. 0.5 mg/mL of Matrigel (BD Biosciences, San Jose, CA, USA) was coated onto the top of the Boyden chamber and incubated at 37 °C for 4 h. After the solidification of Matrigel 5 × 10^4^ cells/well of cells were added to the upper chamber with serum-free media. In total, 500 μL of RPMI media with 20% FBS was added to the lower chamber as a chemoattractant and incubated for 24 h at 37 °C in a CO_2_ incubator. After incubation, the lower part of the upper chamber was permeabilized with 4% formaldehyde and stained with 4′,6-diamidino-2-phenylindole (DAPI). After staining, the cells were visualized under a fluorescent microscope and counted with the use of ImageJ.

### 4.5. Migration Assay

The cells were grown in 6 well plates to 100% confluent monolayer and then scratched with 1 mL sterile pipette tip to form a “wound”. After the wound formation, the cells were incubated in serum-free media for 0, 18, and 24 h at 37 °C in a CO_2_ incubator. The scratch was viewed using an Olympus photomicroscope.

### 4.6. Colony Formation Assay

The cells were seeded in a 6 cm dish with the cell seeding capacity of 500 cells/plate and starved for 12 h in serum-free RPMI media. After 12 h, the serum-free media was discarded and RPMI with 10% heat-inactivated FBS was added. The cells were incubated for 10 days. The media was replaced every 3 days. After 10 days the plates were washed with 1× PBS and then the cells were fixed with 4% formaldehyde for 30 min. After fixation, the cells were stained with 0.6% Giemsa stain for 30 min. The stain was washed with distilled water and then the pictures were taken using a camera.

### 4.7. Gelatin Zymography

To perform gelatin zymography, the cells were seeded in a 6-well plate with the seeding density of 3 × 10^5^ cells/well and incubated at 37 °C in a CO_2_ incubator for 24 h. After incubation, the media were removed and serum-free RPMI media was added. After 12 h, the media was collected in an Eppendorf and centrifuged at 13,000 RPM for 10 min. After centrifugation, the supernatant was resolved in 12% polyacrylamide gel containing gelatin (1 mg/mL). The gels were washed with 2.5% of Triton X-100 for 1 h and then incubated in activation buffer (50 mM Tris–HCl, pH 7.5, 10 mM CaCl_2_) for 16 h at 37 °C. After incubation in the activation buffer, the gels were stained with a staining solution containing 10% glacial acetic acid, 30% methanol, and 1.5% Coomassie brilliant blue for 1 h. After washing, the gels revealed the white lysis zones indicating gelatin degradation, showing the status of MMP-9 and MMP-2.

### 4.8. Western Blot Analysis

The cells were seeded in a 10 cm dish with a seeding density of 2.2 × 10^5^ and incubated at 37 °C in a CO_2_ incubator for 48 h. The cells were collected with the use of a cell scraper and centrifuged at 2000 RPM for 5 min. After centrifugation, the media was removed and centrifuged again to remove the excess media. The pellet was lysed in 500 μL of 2× sample buffer which contains 100 mM of Tris-Cl (pH 6.8), 4% (*w/v*) sodium dodecyl sulphate (SDS), 0.2% (*w/v*) Bromophenol blue and 200 mM of DTT (dithiothreitol). The protein lysates were collected in the Eppendorf tubes and heated at 100 °C for 10 min. The protein was then quantified using the Bradford assay. In total, 30 μg of protein was resolved in 8–12% SDS-PAGE and transferred to methanol-activated PVDF membrane. After transfer, the membrane was blocked with 3% skimmed milk for 15 min and then incubated with specific antibodies for 16 h in 4 °C with 3% skimmed milk in TBST. After incubation, the membrane was washed thrice with TBST each wash for about 10 min followed by the incubation with 1:2000 dilution of horseradish peroxidase (HRP)-conjugated secondary antibody for 1 h in room temperature. The membranes were later washed with TBST buffer three times (10 min/wash) subsequently developed with ECL (electrochemiluminescence) solutions (Bio-Rad Laboratory, Hercules, CA, USA).

### 4.9. Statistical Analysis

The results were expressed as means ± SEM from at least five independent experiments. Significant differences were determined by the one-way analysis of variance (ANOVA) with post-test Newman–Keuls for comparison of at least five treatment groups and Student’s *t*-test for two groups. Statistical significance was defined as *p* < 0.05.
